# The impact of educational interventions on the competence of nurses and midwives in neonatal resuscitation in sub-Saharan Africa: A systematic review

**DOI:** 10.4102/jphia.v16i1.1326

**Published:** 2025-05-15

**Authors:** Andy Emmanuel, Israel Gabriel, Danjuma Aliyu

**Affiliations:** 1 Queensland Ambulance Service, Brisbane, Australia; 2 Institute of Health and Management, Sydney, Australia; 3 Perioperative School, Ahmadu Bello University Teaching Hospital, Zaria, Nigeria

**Keywords:** neonatal resuscitation, educational interventions, competence, nurses/midwives, low-income countries, sub-Saharan Africa

## Abstract

**Background:**

Neonatal mortality is still a significant global public health issue and most of these deaths occur in sub-Saharan Africa. Despite extensive government and nongovernment campaigns, the neonatal fatality rate in this region remains unacceptable.

**Aim:**

This review evaluates the efficacy of educational resuscitation interventions on the knowledge and skills of nurses and midwives about newborns resuscitation.

**Setting:**

Knowledge and skills of nurses and midwives about newborns resuscitation in sub-Saharan Africa.

**Method:**

The review followed Preferred Reporting Items for Systematic Review and Meta-Analyses (PRISMA) standards and used the Grading of Recommendations, Assessment, Development and Evaluation (GRADE) system to evaluate the quality of evidence from the included studies. A search was conducted across seven databases from 2000 to 2024. A cumulative number of 912 studies were retrieved. The review protocol was registered in PROSPERO (CRD42022332734).

**Result:**

The final selection comprised 16 articles. An average grading score of 2.4, suggesting low to moderate evidence. The programmes included the Basic Emergency Obstetrics and Newborn Care training, the Helping Babies Breathe (HBB), the UK Resuscitation Guidelines, the American Heart Council Guidelines, the American Neonatal Resuscitation Program and the Safe Delivery Application. The intervention resulted in considerable improvements in resuscitation knowledge and skills.

**Conclusion**: This review has demonstrated the importance of providing nurses and midwives with training in neonatal resuscitations, as well as the substantial impact it has on the reduction of neonatal mortality rates.

**Contribution:**

This study highlights the need for high-quality data and prioritise locally and culturally acceptable interventions to reduce neonatal mortality in sub-Saharan Africa.

## Introduction

Neonatal mortality is a major global public health issue.^1,2^ In 2021, with a global average death rate of 37 deaths per 1000 live births,^3^ underscoring the critical importance of the neonatal phase, the first 28 days of a child’s life, for survival.^4^ According to the World Health Organization, 2.3 million newborns died within the first 20 days of life globally in 2022, equating to approximately 6500 neonatal deaths daily.^3,5^ The preponderance of neonatal deaths occurs in sub-Saharan Africa, where the incidence is 43% higher than the global average. Approximately, 75% of all neonatal deaths occur within the initial 7 days of life, with approximately one million infants dying on the very day of their birth.^6,7^

Many countries in Africa face the potential of not achieving the Sustainable Development Goals (SDGs) target of reducing the neonatal mortality rate to 25 or less per 1000 live births by 2030.^8^ Thus, collective efforts are vital and greater attention must be directed towards the African continent to implement the necessary actions to minimise the factors contributing to neonatal death, if the SDGs’ targets are to be met. The main factors contributing to neonatal mortality include birth asphyxia, preterm birth, perinatal complications and neonatal sepsis.^9,10^ Birth asphyxia alone was responsible for approximately a quarter of all infant mortality, with 98% of these deaths occurring in low-resource settings.^11,12^

Resuscitation is a proven, evidence-based intervention, necessary for immediate treatment of birth asphyxia and the reduction of infant mortality.^13^ Regrettably, most countries in sub-Saharan Africa either lack the necessary personnel to effectively administer neonatal resuscitation or do not possess the requisite level of competence for its administration.^13,14^ Healthcare professionals frequently demonstrate varying degrees of practical competence in neonatal resuscitation. Factors such as experience levels, resources and access to training can influence this variance.^15,16^ A systematic review and meta-analysis by Patel et al.^17^ demonstrated that training healthcare professionals involved in the delivery process and newborn handling in neonatal resuscitation protocols significantly reduces mortality and morbidity. This suggests that mitigating birth asphyxia by providing resuscitation equipment and ensuring comprehensive training for healthcare professionals in neonatal resuscitation will reduce the neonatal mortality rate in sub-Saharan Africa, consequently, decreasing the global neonatal mortality rate.^18,19^ However, there is a paucity of documented information on neonatal resuscitation practices employed in sub-Saharan Africa and their efficacy.

Thus, the review aimed to assess the effectiveness of educational resuscitation interventions on nurses’ and midwives’ knowledge and skills in neonatal resuscitation in sub-Saharan Africa.

## Methods

### Protocol and registration

This review was conducted in accordance with the protocols outlined in the Preferred Reporting Items for Systematic Reviews and Meta-Analyses (PRISMA) guidelines (Online Appendix 1).^20^ The methodological approach comprised three key components: the search strategy, the selection of relevant articles and the critical evaluation of their methodological quality. This systematic review protocol was registered with Prospective Register of Systematic Reviews (PROSPERO) (CRD42022332734).

### Eligibility criteria

The eligibility criteria for the study were established based on the PICO (patient/population, intervention, comparison, outcome) framework. The population comprised healthcare professionals, specifically midwives and/or nurses. The intervention involved various types of educational resuscitation programmes. Comparisons were made with or without a control group. The outcome measured was competency in neonatal resuscitation. The study design included intervention studies, observational studies (such as cross-sectional studies), single-group studies, two-group studies, experimental studies, quasi-experimental studies and pre- and post-assessments (see Table 1).

**TABLE 1:** Inclusion and exclusion criteria.

Variable	Inclusion criteria	Exclusion criteria
Study design	Intervention studies One group, two groups, experimental, quasi-experimental, pre- and post-assessment	Unpublished studies Qualitative design only Non-English articles
Population	Nurses and/or midwives were involved in the studies.	Studies that were exclusively focused on traditional birth attendants, physicians or other healthcare professionals.
Intervention	Educational resuscitation interventions of any kind	No standard resuscitation programmes such as HBB, Basic Emergency Obstetrics and Newborn Care training, UK Resuscitation Council Manual, American Neonatal Resuscitation Programme, American Heart Association Guidelines were used in the interventions.
Outcomes	Nurses and/or midwives’ competency in neonatal care	Outcomes that did not include the competency of nurses and/or midwives, as well as an emphasis on neonatal care.

HBB, helping babies breathe; UK, United Kingdom.

### Information sources

PubMed, Medline via Ovid, Embase via Ovid, Cumulative Index to Nursing & Allied Health (CINAHL) via EBSCOhost, Web of Science, Cochrane Library and PsycINFO were searched for original articles. The search was limited to English-language scientific articles from 2000 to 2024. We conducted manual searches of abstracts from reference lists of pertinent reviews to find any additional studies.

### Search strategy and study selection

To effectively combine Medical Subject Headings (MeSH) terms and keywords in a search, we firstly identified relevant MeSH terms in the *PubMed* and *Medline* and then integrated them with keywords using Boolean logic operators. The search terms consisted of various combinations of the following keywords: impact; effect; influence and educational intervention; nursing intervention; neonatal resuscitation; supportive education; resuscitation training; therapy; treatment; strategies and competency; competence and healthcare professionals; health workers; nurses; midwives and neonatal care; infant care; intrapartum care and low-income countries; sub-Saharan Africa; middle- and low-income countries; developing countries. The search strategy was modified as appropriate for different databases.

**FIGURE 1:**
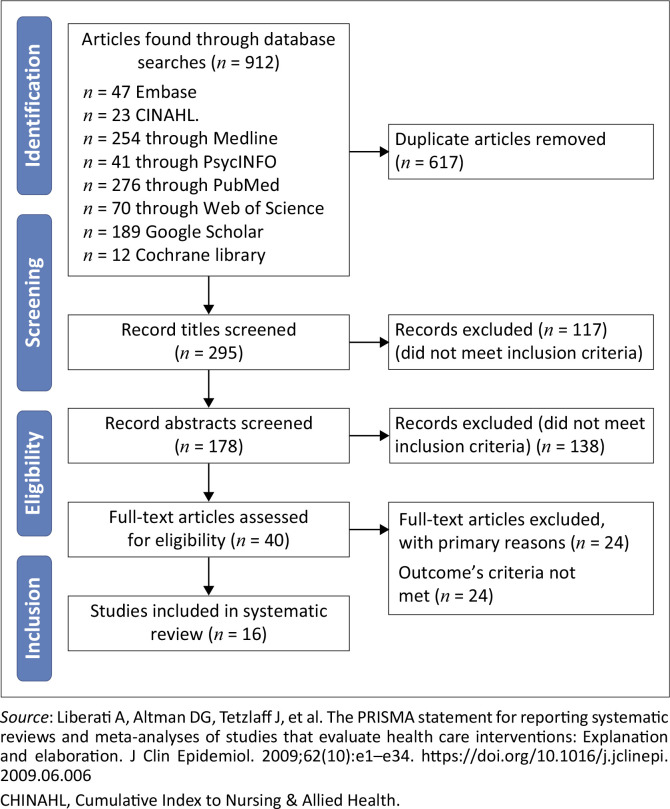
Preferred Reporting Items for Systematic Reviews and Meta-Analyses (PRISMA), 2020 flow diagram for systematic reviews of the effects of educational interventions on the competency of nurses and midwives in neonatal resuscitation.

A total of 912 articles were downloaded and exported to reference management software using the latest version of EndNote (version 21), which was used to remove the duplicate articles. The titles and abstracts of the remaining articles were subsequently screened. The articles that failed to meet the eligibility criteria were excluded. The remaining articles were reviewed based on the inclusion and exclusion criteria, resulting in the selection of 16 articles for comprehensive review, as illustrated in the PRISMA flowchart (see Figure 1), and subsequently evaluated by two reviewers (A.E. and I.G.). The main author (A.E.) and second reviewer (I.G.) double-checked all the retrieved data. Each study was initially assessed based on its title by A.E. and I.G., followed by an independent review of all the abstracts and full-text records by A.E. and I.G. separately. Following this process, the eligible studies were considered for inclusion via Covidence. Initial disagreements regarding 14 articles were resolved through consensus-building or adjudication by a third reviewer (D.A.).

### Data extraction and data items

All pertinent data were gathered and entered into a Microsoft Excel spreadsheet. All retrieved data were double-checked by the main reviewer (A.E.), then by the second and third reviewers (I.G., D.A.). Included studies featured over 70% of nurses and/or midwives employed in clinical settings who demonstrated knowledge and competencies in neonatal care. Qualitative design studies were excluded because of the focus on a quantitative systematic review.

The data extracted comprised general information (first author, study location, publication year, objectives), methodological details (study design, sample context, participants, intervention, mode of delivery, content and measurement tools) and study findings (quantitative outcomes) (see Table 2).

**TABLE 2:** Grading of included articles based on quality of evidence.

Articles	Limitation	Imprecision	Indirectness	Reporting bias	Grade
Gomez et al.^26^	Risk of bias was significant. Participants were aware which group of the intervention they belonged to, and outcome of interest was known by various facilities and participants therefore may potentially affect the quality of data.	Robust statistical analysis to determine power.	Yes Recommendations were directed to participants studied.	Publication bias may be eminent because authors did not report the total number of maternity health workers and what percentage of the participants was the 403 that signed up for the study.	3
Umoren et al.^27^	Study was limited to secondary and tertiary facilities.	Good	No. Finding may differ from participants in rural settings of primary facilities.	None identified	3
Odongkara et al.^28^	Intra-cluster variation was identified and this is associated with inadequate sample size.	Poor because of inadequate sample size.	Participants were same with those for whom the recommendation applies	None identified	3
Lund et al.^29^	Facilities rather than participants were randomised, blinding was not possible and loss of 26% of health workers that stated the study. It was unclear how the 176 health workers were arrived at. Conclusion only applies to the place of study. Conclusion not externally valid.	Estimate may not be precise because of the limited sample size.	Appropriate	None identified	3
Umar et al.^30^	No control, weak design, no report about how instrument was validated.	Sample size and power analysis was not performed.	Recommendation directed at participants	None	2
Nyiringango et al.^31^	No control, presence of cofounders such as participating in another training was not determined.	Sample size and power analysis were performed.	Recommendation directed at participants	None	2
Enweronu-Laryea et al.^32^	No control group	Sampling size and power analysis not reported.	Directed at study population and wider population	Possible	2
Ding et al.^33^	No control, small sample	Sampling size and power analysis not reported.	Good and directed at current population	Nil	2
Opiyo et al.^34^	There was limited success in randomisation. There were chances of cross-group contamination and observer bias related to lack of observer blinding.	Sampling size and power analysis reported.	Good and directed at current population	Nil	3
Ameh et al.^35^	No control	Sampling size and power analysis not reported.	Good and directed at current population	Nil	2
Makene et al.^36^	No control	-	Good and directed at current population	Nil	2
Trevisanuto et al.^37^	No control	-	Good and directed at current population	Nil	2
Wilson et al.^38^	No control	-	Good and directed at current population	Nil	2
Draiko et al.^39^	No randomisation	Sampling size and power analysis performed with limitation because of war.	Good and directed at current population	Nil	2
Nishimwe et al.^40^	Small sample	Sampling size and power analysis not reported.	Good and directed at current population	Nil	3
Umunyana et al.^41^	No control, small sample	Sampling size and power analysis not reported.	Good and directed at current population	None	2

Note: Please see the full reference list of the article Emmanuel A, Gabriel I, Aliyu D. The impact of educational interventions on the competence of nurses and midwives in neonatal resuscitation in sub-Saharan Africa: A systematic review. J Public Health Africa. 2025;16(1), a1326. https://doi.org/10.4102/jphia.v16i1.1326, for more information

### Quality of study

The Grading of Recommendations, Assessment, Development and Evaluation (GRADE) system was employed to evaluate the quality of evidence obtained from the included studies. The GRADE approach provides a systematic framework for evaluating the certainty of evidence in systematic reviews.^21,22,23^ The quality of evidence was determined using five domains: study limitations or risk of bias, inconsistency of results, indirectness of evidence, imprecision and reporting or publication bias^22,24^ (see Table 2).

The GRADE system categorised evidence into four distinct levels, also known as certainty in evidence or quality of evidence. The levels comprised very low, low, moderate and high.^25^ Guyatt et al.^22^ indicate that high-quality evidence (Grade 4) entails further research is very unlikely to change our confidence in the estimated effect. In contrast, further research is likely to have an important impact on our confidence in the estimate of effect and may change the estimate for moderate quality (Grade 3). For evidence of low quality (Grade 2), further research is very likely to have an important impact on our confidence in the estimate of effect and may change the estimate. Evidence of very low quality (Grade 1) means that any estimate of effect is highly uncertain. In addition, the strength of recommendation can be categorised as either weak or strong (see Table 3).^22^

**TABLE 3:** Key aspects of the GRADE system.

Grading	Description
Grade 1	Very low-quality evidence means any estimate of effect is very uncertain.
Grade 2	Low-quality evidence: Further research is very likely to have an important impact on our confidence in the estimate of effect and may change the estimate.
Grade 3	Moderate quality evidence: Further research is likely to have an important impact on our confidence in the estimate of effect and may change the estimate.
Grade 4	High-quality evidence: Further research is very unlikely to change our confidence in the estimated effect.

Source: Guyatt GH, Oxman AD, Vist GE, et al. GRADE: An emerging consensus on rating quality of evidence and strength of recommendations. Br Med J. 2008;336(7650):924–926. https://doi.org/10.1136/bmj.39489.470347.AD

**TABLE 4:** Summary of findings from included articles.

Title and authors	Design	Year and place of publication	Population and the sample size	Intervention	Outcome and assessment method	Summary of findings	Comments
Accelerating newborn survival in Ghana through a low-dose, high-frequency health worker training approach: a cluster randomised trial Gomez et al.^26^	RCT	2018 Ghana	403 midwives from 42 facilities	Basic Emergency Obstetrics and Newborn Care (BEmONC) training	Newborn mortality, knowledge, and skill retention after 24 h. OSCE and knowledge test	Sustained decrease in facility-based newborn mortality and intrapartum stillbirths and retained knowledge and skills among SBAs after a year.	Direct observation in a real-life setting will provide a more reliable result.
Adding video debriefing to Helping Babies Breathe (HBB) training enhanced retention of neonatal resuscitation knowledge and skills among healthcare workers in Uganda: A cluster randomised trial Odongkara et al.^28^	RCT	2020 Uganda	86 skilled birth attendants, nurses and midwives	Standard HBB versus HBB plus video debriefing	Knowledge retention/ MCQ & OSCE	Video debriefing had a significant effect on knowledge, skills and knowledge retention.	Intervention based on standard package that requires equipment that may affect sustainability or technology that is expensive to access and sustain in current setting.
Electronic Helping Babies Breathe (eHBB): a randomised controlled trial of virtual reality or video for neonatal resuscitation refresher training in healthcare workers in resource-scarce settings Umoren et al.^27^	RCT	2021 Nigeria and Kenya	274 nurses	eHBB Virtual Reality versus Video versus control group.	Knowledge retention MCQ & OSCE	Digital interventions supported the retention of neonatal resuscitation knowledge and skills for HCWs in Nigeria and Kenya.	Knowledge retention does not necessarily translate into quality care.
Association Between the Safe Delivery App and Quality of Care and Perinatal Survival in Ethiopia: A Randomised Clinical Trial Lund et al.^29^	RCT	2016 Ethiopia	176 Community healthcare workers, nurses, midwives 3601 pregnant women	Safe Delivery Application	Skills and knowledge retention of neonatal resuscitation	The intervention was effective in improving and sustain skills and knowledge.	Blinding was not possible and facilities rather than participants were randomised. Conclusion not externally valid.
Evaluation of the cognitive effect of newborn resuscitation training on healthcare workers in selected states in Northern Nigeria. Umar et al.^30^	Pre- and post-intervention cross-sectional study	2018 Nigeria	293 healthcare workers, including nurses, midwives and doctors	3–6 days implementation of UK Resuscitation Council Manual	Written test before and after intervention	Knowledge of healthcare workers improved in the post-training period.	There was no information about how the instrument used for data collection was validated. Sample size and power analysis was not performed or reported.
Assessing the impact of mentorship on knowledge about and self-efficacy for neonatal resuscitation among nurses and midwives in Rwanda Nyiringango et al.^31^	Pre- and post-quasi experimental	2021 Rwanda	123 Nurses	Two weeks programme on emergency obstetric. including neonatal resuscitation	Written test before and after intervention	Knowledge and self-efficacy improved	Possible presence of confounders such as participating in another training was not assessed.
Evaluating the effectiveness of a strategy for teaching neonatal resuscitation in West Africa Enweronu-Laryea et al.^32^	Pre- and post-intervention cross-sectional study	2009 Ghana	271 Nurses, midwives, nurse anaesthetist and physicians	Content from American Neonatal Resuscitation programme was adapted with in-depth description of the seven subjects in the module for 2 to 3 days	Written test before and after intervention	Sustaining evidence-based Neonatal Resuscitation Training (NRT) programme is feasible in West Africa.	Power analysis not performed. Recommendation generalised
Evaluation of a Neonatal Resuscitation programme for healthcare professionals in Zanzibar Tanzania: a pre- and post-intervention study Ding et al.^33^	Pre- and post-intervention cross-sectional study	2021 Tanzania	23 Nurses, midwives and physicians	Two days training guided by the American Heart Association Guidelines	Written and practical (observation) tests before and after the study	Knowledge and skill improved after training	Small sample
Effect of Newborn Resuscitation training on healthcare workers’ practices in Pumwani hospital, Kenya Opiyo et al.^34^	RCT	2008 Kenya	83 Nurses and midwives	One-day training adapted from UK Resuscitation Council	Compared practical skills among two groups	There was significant improvement in practices and reduction in frequency of inappropriate and potentially harmful practices.	Design and implementation appear good, however, there was limited success in randomisation. There were chances of cross-group contamination and observer bias related to lack of observer blinding.
Knowledge and skills of healthcare providers in sub-Saharan Africa and Asia before and after competency-based training in emergency obstetric and early newborn care Ameh et al.^35^	Pre- and post-intervention cross-sectional study	2016 2 African countries and 7 Asian	5939 Doctors, midwives, physicians and nursing aides	Emergency Obstetric Care (EmOC) training package was used for 3–5 days.	Knowledge was assessed using questionnaires, while skills were assessed using scenario-based assessment and OSCE.	There was a significant improvement in knowledge and skills after receiving a short competence-based training in emergency obstetric and early newborn care.	No control, power analysis not reported.
Improvements in newborn care and newborn resuscitation following a quality improvement programme at scale: Results from a before and after study in Tanzania Makene et al.^36^	Pre- and post-intervention cross-sectional study	2014 Tanzania	489 Newborns were observed in the pre-2010 and 560 in the post-intervention, 206 in 2010 and 217 in 2012 for knowledge assessment, while 299 in 2010 and 213 in 2012 for skill assessment (simulation)	Emergency Obstetric Care training package was used.	Knowledge was assessed using questionnaires, while skills were assessed using standard checklist.	There was a significant improvement in essential newborn care.	Skills assessment can be better assessed through direct observation.
Effect of a Neonatal Resuscitation course on healthcare providers’ performances assessed by video recording in a low-resource setting Trevisanuto et al.^37^	Pre- and post-intervention cross-sectional study	2015 Mozambique	100 Nurses and midwives	Neonatal Resuscitation programme adapted from American Heart Association Guidelines in 1-day training	Performance was assessed using video recording.	There was a significant improvement in the performance score.	Weak design
Helping Babies Breathe implementation in Zanzibar, Tanzania Wilson et al.^38^	Correctional study with data collection at 3 time points	2017 Tanzania	33 Midwives	HBB in 3-day training	Knowledge was assessed using questionnaire, performance was assessed using structured observation and OSCE. Interviews were also conducted before and after the study.	Knowledge and skills improved and were sustained.	Weak design and low sample may result in bias.
Knowledge, skills and competency retention among healthcare workers 1 year completing Helping Babies Breathe training in South Sudan Draiko et al.^39^	Non-randomised pre- and post-controlled group	2019 South Sudan	123 Healthcare workers comprising doctors, nurses, midwives, community healthcare workers and skilled birth attendants	HBB a day	Knowledge questionnaire and direct observation using OSCE	There was a significant improvement in knowledge and the performance score. There is a marked decline in knowledge and skill score over time.	Fair design, moderate evidence depending on study limitation. A strong design will generate stronger evidence.
The effect of and learning application on nurses’ and midwives’ knowledge and skills for the management of postpartum haemorrhagic (PPH) and neonatal resuscitation: Pre- and post-intervention study Nishimwe et al.^40^	Pre- and post-intervention cross-sectional study	2021 Rwanda	54 Nurses and midwives	Safe delivery application using basic emergency obstetrics and neonatal care using a smart phone	Knowledge questionnaire and scenario-based simulation	Safe delivery application significantly improved knowledge and skills of nurses and midwives on management of PPH and neonatal resuscitation as long as for 6 months.	Low sample and weak design
A practice improvement package at scale to improve management of birth asphyxia in Rwanda: A before-and-after mixed methods Umunyana et al.^41^	Pre- and post-intervention mixed methods without control group	2020 Rwanda	220 Midwives and nurses	HBB for 5 days	Knowledge was assessed using questionnaire and performance was assessed using HBB OSCE tool.	Knowledge and skills improved with enhanced clinical practices and also improvements observed in the newborns’ outcomes.	Weak design

Note: Please see the full reference list of the article Emmanuel A, Gabriel I, Aliyu D. The impact of educational interventions on the competence of nurses and midwives in neonatal resuscitation in sub-Saharan Africa: A systematic review. J Public Health Africa. 2025;16(1), a1326. https://doi.org/10.4102/jphia.v16i1.1326, for more information

HBB, helping babies breathe; MCQ, multiple-choice questions; OSCE, objective structured clinical examination; RCT, randomised controlled trials; SBA, skilled birth attendant; HCW, healthcare worker; UK, United Kingdom.

### Data analysis

The analysis of findings is presented in a narrative format, typically comprising a summary followed by a discussion of the study’s characteristics and results. The Cochrane Collaboration advocates for this approach,^42^ particularly in instances where the data from the included studies exhibit heterogeneity, as observed in this study, which made it impossible to combine the findings of the meta-analysis. A narrative depiction is provided as an overview of educational resuscitation interventions affecting healthcare professionals’ competency in neonatal care. The participants of the study are characterised and their outcomes are presented using descriptive statistics.

## Results

### Summary of included studies

The outcomes of the quality assessment process are reported in accordance with the standards established by PRISMA. This includes the selection of studies, characteristics of the studies, assessment of bias risk and the findings. Three studies were conducted in Rwanda^31,40,41^ and three studies in Tanzania.^33,36,38^ Two studies were conducted in Nigeria,^27,30^ two in Kenya^27,34^ and two in Ghana.^26,32^ A study was conducted in each of four countries: Uganda,^28^ Ethiopia,^29^ South Sudan^39^ and Mozambique.

### Risk of bias

The GRADE system assessment of bias risk revealed that none of the studies fulfilled the criteria for high-quality evidence (Grade 4). Table 2 indicates that all studies were assessed as having moderate-quality evidence (Grade 3) or low-quality evidence (Grade 2). Six studies^26,27,28,29,34,40^ were rated as moderate quality, while 10 studies were categorised as low quality (see Table 2).^30,31,32,33,35,36,37,38,39,41^

The poor quality of the studies was associated with selection issues, outcome measures that predominantly depended on self-reporting by participants and the characteristics of the interventions used in the randomised controlled trials (RCTs), which rendered blinding unfeasible. Additional biases encompass limitations such as an exclusive focus on secondary and tertiary institutions, intra-cluster variation, inadequate sample sizes, and the existence of publication bias. Common weaknesses of these studies included confounders such as a high withdrawal or dropout rate, along with the absence of a control group.

The average grading score for the 16 articles is approximately 2.4, indicating that the quality of evidence from these studies ranges from low to moderate. Despite the generally low quality of the studies, their consistent findings contribute valuable insights into the field and have been incorporated into the results.

### Characteristics of included studies

A total of 16 research articles were included in the final selection, with publication years spanning from 2008^34^ to 2021^27,31,33,40^ A total of 5620 nurses and midwives participated in these 16 studies. The articles included five RCTs,^26,27,28,29,34^ two non-randomised controlled^39,40^ and nine before-and-after studies without controlled groups.^30,31,32,33,35,36,37,38,41^ Additional information regarding the included studies is provided in Table 2.

### Type and duration of intervention

The majority of the studies included in this review^26,29,31,35,36,40^ used the Basic Emergency Obstetrics and Newborn Care (BEmONC) training programme as the primary intervention, indicating its widespread use in the research field. Furthermore, a considerable number of studies^27,28,38,39,41^ used the Helping Babies Breathe (HBB) intervention. Additional training packages utilised included the United Kingdom (UK) Resuscitation Guidelines,^30,34^ and the American Heart Council Guidelines.^32,33,37^ The American Neonatal Resuscitation Programme (ANRP) and Safe Delivery Application were developed in local languages and incorporated the video features to improve healthcare professionals’ abilities in providing high-quality neonatal care. The training duration ranged from 1 day to 2 weeks. The interventions were implemented through various modes of delivery, including telephone, face-to-face and virtual formats.

### Outcome measures

The assessment utilised multiple-choice questions (MCQs), objective structured clinical examinations (OSCEs), baby-mask ventilation and record analysis. Knowledge and skills were evaluated immediately following training and subsequently to assess retention. Several studies assessed the impact of training on neonatal survival rates. The effect of the training programme on neonatal survival was evaluated by Gomez et al.^26^ While Odongkara et al.^28^ and Umoren et al.^27^ assessed knowledge retention after training, Gomez et al.^26^ determined how the intervention reduced the newborns’ mortality, improved knowledge about neonatal resuscitation and skills retention after 24 h. Similarly, Lund et al.^29^ evaluated skills and knowledge regarding neonatal resuscitation following the intervention. Refer to Table 2 for additional information.

This finding indicates variation in the outcome of interest among the authors of the included studies. Most studies focused on the effects of training on acquired knowledge and skills, while others examined its impact on the decrease in neonatal mortality.

### Impact of interventions

The main outcomes of the included studies varied slightly; however, the findings indicate an overall improvement in the assessed parameters. Gomez et al.^26^ found that participants decreased facility-based newborn mortality and intrapartum stillbirths, while maintaining knowledge and skills after a year. Specifically, after 1 year, skilled participants exhibited scores that were 28% (95% confidence interval [CI]: 25–32; *p* < 0.001) and 31% (95% CI: 27–36; *p* < 0.001) higher than baseline. Umunyana et al.^41^ found that the training had a positive effect on knowledge, skills, clinical practices and newborn outcomes. The 6-month HBB training and mentorship programme resulted in a significant reduction in neonatal admissions because of asphyxia, decreasing from 22.0 per 1000 live births in 2015 to 13.9 in 2018. The annual reduction was 12.9% (95% CI: 8.3% – 17.5%). Neonatal deaths attributed to asphyxia in health facilities declined from 3.8 deaths per 1000 live births in 2015 to 3.3 deaths per 1000 live births in 2018, indicating an annual reduction of 4.2% (95% CI: 0.2% – 6.3%).

Odongkara et al.^28^ concluded that video debriefing significantly impacted knowledge, skills (adjusted mean difference: 5.34; 95% CI: 0.82–10.78), and knowledge retention (adjusted mean difference: 2.97; 95% CI: 1.52–4.41). Umoren et al.^27^ found that digital interventions enhanced the retention of neonatal resuscitation knowledge and skills among healthcare workers in Nigeria and Kenya. Specifically, the retention of bag-mask ventilation (BMV) skills at 6 months was greater in the virtual reality (VR) group (−15% VR, *p* = 0.10) compared to the video group (−21%, *p* < 0.01) and the control group (−27%, *p* = 0.001). Lund et al.^29^ found that the intervention effectively improved and sustained healthcare workers’ knowledge and skills in neonatal resuscitation. Intervention skill scores among healthcare workers showed a significant increase compared to the control group at the 6-month mark. The mean difference at 6 months was 6.04 (95% CI: 4.26–7.82), and at 12 months, it was 8.79 (95% CI: 7.14–10.45), compared to 80%. Other studies indicated an enhancement in knowledge and/or performance regarding neonatal resuscitation.^34^

Perfect resuscitation was observed in 28.0% of cases with a relative risk of 2.60 (95% CI: 1.53–4.43, *p* < 0.001), while adequate resuscitation occurred in 68.1% of cases with a relative risk of 2.22 (95% CI: 1.64–2.99, *p* < 0.001). Ding et al.^33^ reported a mean difference of –4.00 (95% CI: −5.900 to −2.099, *p* < 0.001). Enweronu-Laryea et al.^32^ also found significant results (*p* < 0.001), as did Makene et al.^36^ The knowledge of healthcare workers, as demonstrated through a case study, increased significantly from 23% to 41% (*p* < 0.0001). Additionally, Wilson et al.^38^ reported a significance level of *p* < 0.05. Ameh et al.^35^ observed an improvement of 10.0% (5.0% – 15.0%) in knowledge and 28.8% (23.1% – 35.1%) in skill. Further studies by Umar et al.^30^ and Nishimwe et al.^40^ also contribute to this body of evidence. The mean difference for NR knowledge is 19.1 (95% CI: 16.31–21.76), while for NR skills, it is 5.5% (95% CI: 3.66–7.41). Nyiringango et al.^31^ reported a significant increase in participants’ knowledge from pre-test to post-test, with a mean difference of 15.17 (95% CI: 12.8–17.6). The self-efficacy for neonatal resuscitation exhibited a significant increase from pre-test to post-test mean (Mean difference = 2.17, 95% CI: 1.849–2.505) among nurses and midwives. Draiko et al.^39^ found that immediate evaluation of healthcare workers following Helping Babies Breathe training led to a significant increase in knowledge, skills and competency in neonatal resuscitation.

However, this improvement declined over 1 year, with a mean difference increase of 55.2 (50.9–59.6, *p* < 0.05). A slight decrease was observed between the immediate post-intervention and the 3-month follow-up, with a mean difference of 13.3 (–17.7 to 8.87, *p* < 0.05). The 1-year mean skill scores decreased from 94.5% to 77% following the 3-month assessment. Trevisanuto et al.^37^ noted that while resuscitations did not meet the recommended standards for quality and execution time, the clinical practice of healthcare providers improved following their participation in an adapted NRP course. Resuscitation scores exhibited a notable enhancement across all levels following the course, with the score for ‘initial steps’ rising from 33% to 44%.

## Discussion

Birth asphyxia constitutes a significant contributor to global infant mortality rates. Birth asphyxia is responsible for approximately one million neonatal deaths worldwide each year.^43^ Improving the availability of accurate and reliable data is essential for reducing preventable newborn mortality, as it supports the development of evidence-based interventions through research and development initiatives. There remains considerable work to be done in this area, especially in sub-Saharan Africa, which continues to experience the highest rates of infant mortality. This study offers a comprehensive overview of research on the impact of neonatal resuscitation training on infant survival rates. The research findings provide a foundational basis for future high-quality studies aimed at generating robust data on the efficacy of birth asphyxia therapy in reducing infant mortality.

The implementation of a grading system for assessing the quality of evidence from research conducted in sub-Saharan Africa has enabled a systematic and fair method for evaluating the value of these studies. The analysis encompassed research that examined the impact of neonatal resuscitation training on participants’ knowledge, skill proficiency and infant survival rates. Nonetheless, the information currently available on this matter is typically classified as having poor to intermediate quality. Only one study has specifically investigated the effects of training on newborn survival. Therefore, it is essential to investigate the impact of neonatal resuscitation training on healthcare professionals’ performance and newborns survival rates. Including data on participants’ satisfaction with the training, their acquisition of knowledge and skills and the subsequent impact on neonatal care quality, as well as the reduction of neonatal mortality and morbidity, would have provided greater insight.

Another significant observation was the use of various interventions across the included studies, all of which were identified as externally sourced rather than locally produced. Table 2 illustrates that the training programmes for resuscitation included several components: basic emergency obstetric and newborn care training, the use of HBB (sometimes enhanced with video debriefing), the application of the Safe Delivery App, consultation of the UK Resuscitation Council Manual, incorporation of the American Neonatal Resuscitation Programme and compliance with the guidelines from the American Heart Association. Regions with elevated infant mortality rates should consider the localisation or adaptation of current programmes, as many initiatives were initially designed for resource-rich and technologically advanced environments. Cultural influences may significantly affect the efficacy of certain programmes. To achieve broad acceptability of an intervention, it is essential to consider factors including the surrounding environment, cultural appropriateness and long-term viability.

Our analysis of the publications in the review revealed a lack of studies offering high-quality data. This finding highlights the need for enhanced investment in the development and implementation of newborn resuscitation therapies tailored to the specific needs of sub-Saharan Africa. Expeditious execution of rigorous investigations, including experimental or quasi-experimental studies, is essential in this field to generate robust evidence for preventing infant mortality because of asphyxia. There is an urgent need to implement regular professional development activities aimed at enhancing the capacity of healthcare workers to effectively perform neonatal resuscitation, particularly in sub-Saharan Africa, where a significant number of these deaths occur. A recent report indicated that sub-Saharan Africa has the highest neonatal mortality rate, accounting for 43% of global neonatal deaths. The review indicates a scarcity of high-quality studies that provide the necessary evidence to enhance neonatal care in low-income countries and to meet the SDGs. Consequently, substantial investment in local research is essential to produce high-quality evidence.

The World Health Organization, in collaboration with the Ministries of Health, has prioritised strategies such as midwife-led continuity of care, where the same midwives provide care to women from the antenatal to postnatal periods. This approach has effectively reduced pre-term births. There is a necessity for ongoing professional development programmes for midwives and nurses, along with continuous evaluation of intervention impacts to monitor the progress and identify the most effective strategies for scaling up. Educational programmes are likely to achieve greater effectiveness when designed with high-quality, localised data.

### Strengths and limitations

The strength of this study is in the quality of the frameworks employed to evaluate the articles included in this review. The PRISMA guideline and GRADE system provide a foundation for systematic and objective evaluations. Thus, lending legitimacy to the evaluation process.

The moderate quality of the studies included in this review is the primary limitation, since it undermines the quality of evidence and the robustness of any recommendations derived from the presented findings. This emphasises the necessity for high-quality research in sub-Saharan Africa to produce robust data on sustainable interventions aimed at lowering infant mortality.

## Conclusion and recommendation

The study revealed that training healthcare professionals in newborn resuscitation may significantly reduce neonatal or infant mortality in sub-Saharan Africa. The various training approaches employed in the studies demonstrated a substantial improvement in newborns survival rates.However, the review highlighted a shortage of research with high-quality data in sub-Saharan Africa. The primary treatments examined in the evaluated research studies were developed outside the sub-Saharan region and may not have adequately accounted for cultural and environmental factors. To achieve the SDGs of reducing global neonatal mortality, it is imperative to conduct studies that generate high-quality data in regions with high neonatal mortality rates such as sub-Saharan Africa, while emphasising interventions that are culturally and locally acceptable. Governments in countries with high infant mortality rates may significantly reduce these rates by investing in researching and formulating regulations that enhance healthcare personnel’s capacity to manage neonatal hypoxia and other critical conditions.
